# Research on the Classification of Traditional Building Materials in Southern Fujian Using the Reflection Intensity Values of Ground-Based LiDAR

**DOI:** 10.3390/s25020461

**Published:** 2025-01-15

**Authors:** Tsung-Chiang Wu, Neng-Gang Kuan, Wei-Cheng Lu

**Affiliations:** Department of Civil Engineering and Engineering Management, National Quemoy University, Kinmen 89250, Taiwan; kmit313773gis@gmail.com (N.-G.K.); eric.w.c.lu@gmail.com (W.-C.L.)

**Keywords:** automated classification, laser reflectance intensity (I-value), boxplot, polynomial regression curve, attribute function equation

## Abstract

Ground-based LiDAR technology has been widely applied in various fields for acquiring 3D point cloud data, including spatial coordinates, digital color information, and laser reflectance intensities (I-values). These datasets preserve the digital information of scanned objects, supporting value-added applications. However, raw point cloud data visually represent spatial features but lack attribute information, posing challenges for automated object classification and effective management. Commercial software primarily relies on manual classification, which is time-intensive. This study addresses these challenges by using the laser reflectance intensity (I-value) for automated classification. Boxplot theory is applied to calibrate the data, remove noise, and establish polynomial regression equations correlating intensity with scanning distances. These equations serve as attribute functions for classifying datasets. Focusing on materials in traditional Minnan architecture on Kinmen Island, controlled indoor experiments and outdoor case studies validate the approach. The results show classification accuracies of 74% for wood, 98% for stone, and 93% for brick, demonstrating this method’s effectiveness in enhancing point cloud data applications and management.

## 1. Introduction

LiDAR (light detection and ranging) technology has been widely applied in various professional fields, providing high-precision, high-density, and visually rich 3D point cloud data, including spatial coordinates and digital color information. Regardless of the application target or domain, the effective management of 3D digital point cloud data is critical to the success of subsequent applications. These applications include digital preservation and structural analysis of traditional architectural heritage, urban 3D modeling for infrastructure management, environmental monitoring for natural disaster risk assessment (e.g., landslides and floods), and high-precision mapping for autonomous driving systems. Efficient classification of 3D point cloud data enhances the accuracy and efficiency of these applications while also expanding their scope and depth.

However, the coordinate information within 3D point cloud data lacks corresponding attribute data to identify which spatial objects the coordinates belong to. While it can realistically represent the 3D spatial appearance of target objects, it fails to provide actionable information for classification. In practice, most currently available 3D point cloud processing software relies on manual visual selection and deletion for data classification. Such methods are labor-intensive and time-consuming, especially for complex data extraction tasks. Automating the classification of point cloud data would streamline and refine processes in existing application domains, as well as expanding potential research and application opportunities.

Traditional methods utilizing geometric shapes and feature extraction have proven ineffective for point cloud data classification. As a result, applying the laser reflectance intensity (I-value) for classifying 3D point cloud models emerges as a promising research direction [1]. Current studies and applications using I-values for 3D point cloud classification have predominantly focused on full-waveform 3D point cloud data acquired from airborne LiDAR. Full-waveform data provide more comprehensive records of the physical properties of reflective surfaces compared with ground-based LiDAR [2,3].

Nevertheless, point cloud data collected via ground-based LiDAR in outdoor settings are inevitably influenced by environmental and physical factors. Environmental factors can be mitigated by conducting scans under consistent atmospheric conditions, while physical factors require consideration of the target material’s surface characteristics, data acquisition geometry, laser reflectivity, scanning distance, and incident angle. Even for surfaces with the same material properties, the I-values exhibit clustering phenomena within a specific range. This clustering behavior strongly correlates with environmental and physical factors during the scanning process, making it a key basis for automated 3D point cloud classification.

This study focused on materials commonly used in traditional Minnan architecture on Kinmen Island, specifically stone, wood, and brick. This study developed a classification framework using I-values from ground-based LiDAR. The process starts by applying boxplot analysis to detect and remove anomalies in I-values for these materials. Regression equations are then established for each material, incorporating environmental and physical factors. These regression equations serve as classification indicators, enabling the automated classification of the three traditional materials in Minnan architecture.

## 2. Materials and Methods

### 2.1. Classification Targets

**Figure 1:**
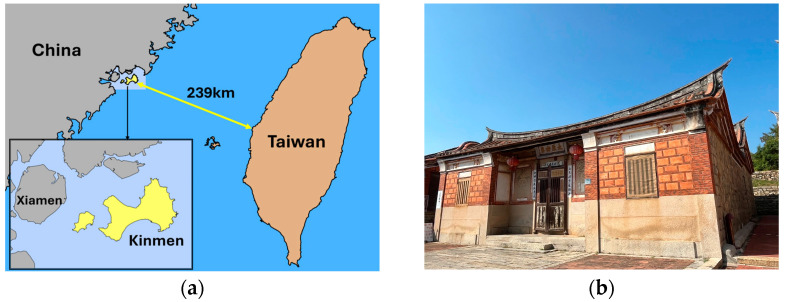
(**a**) Location of Kinmen Island; (**b**) traditional Minnan-style architecture.

Kinmen Island is situated southeast of Fujian, China, approximately 10 km west of Xiamen and 239 km east of Taiwan (Figure 1, left). The island is home to numerous traditional Minnan-style buildings (Figure 1, right), which are considered culturally and historically valuable assets. These Minnan-style buildings predominantly use stone, wood, and brick as their primary construction materials. Therefore, this study selected these three materials as the classification targets.

### 2.2. Ground-Based LiDAR and Laser Reflectance Intensity (I-Value)

This study employed the FARO Focus 3D LiDAR (FARO Technologies Inc., Lake Mary, FL, USA) system to explore the application of the laser reflectance intensity (I-value) from point cloud data acquired via ground-based LiDAR for classification research. This system provided precise laser reflectance intensity (I-value) data, which were essential for analyzing the differences between the materials and constructing the classification model [4,5,6].

The 3D point cloud data obtained via ground-based LiDAR include spatial 3D coordinates and color values as well as the laser reflectance intensity (I-value), which measures the strength of the laser signal reflected back to the scanner after striking the surface of a scanned object. The I-value is influenced by the reflectivity of the scanning target, the distance to the target, and the angle of incidence. Reflectivity, in turn, is affected by factors such as the surface roughness, color, and brightness of the scanned object.

Thus, the I-value recorded via ground-based LiDAR can be considered a function of the target’s reflectivity (ρ), laser incidence angle (θ), and distance (R). The equation for the laser reflectance intensity (I-value) can be expressed as shown in Equation (1) [7,8].

(1) $$I\propto \rho cos\theta R^{-2}$$

The larger the laser reflectance intensity (I-value), the closer the point cloud appears to white; conversely, the smaller the I-value, the closer the point cloud appears to black. When the point cloud appears black, it indicates an invalid reflection, making it impossible to calculate the distance from the scanner to the surface of the scanned object. Consequently, the spatial coordinate of that point cannot be obtained. This demonstrates that the laser reflectance intensity (I-value) can serve as one of the indicators for assessing the quality of scanning data [9].

When a point cloud lacks data due to invalid reflections, forming black patches, this phenomenon is closely related to the physical principles of laser scanning and reflection characteristics. Certain materials, such as deep black or highly absorptive surfaces, absorb most of the laser light, preventing the scanner from receiving sufficient reflected signals to generate data. Similarly, when the laser beam strikes the surface of an object at extreme angles, the reflected beam may fail to return to the scanner, resulting in missing data. Environmental factors, such as ambient light interference, fog, dust, or transparent objects (e.g., glass), can also weaken or scatter the laser beam, further reducing the scanner’s ability to capture return signals. The sensitivity of different laser scanning devices to weak reflections varies. Some may entirely disregard signals below a specific threshold, resulting in “black” or empty areas in the point cloud. The primary reason for the appearance of black patches is a lack of sufficient point data in these regions. This absence of data prevents the system from assigning colors during image texturing or coloring processes, causing these areas to appear black due to their inability to display any meaningful information. This visual characteristic directly reflects the data void in the point cloud.

### 2.3. Boxplot

**Figure 2:**
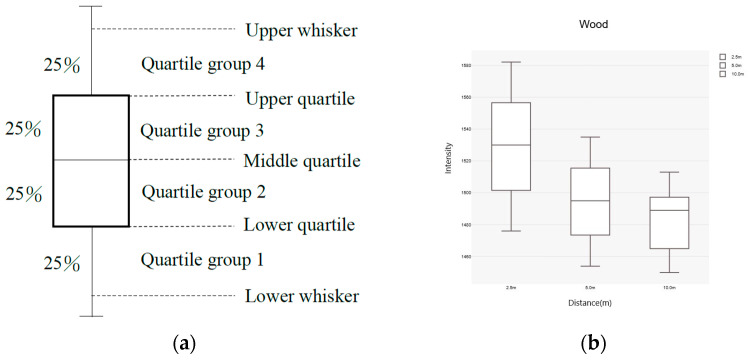
(**a**) Boxplot; (**b**) comparison of boxplots for different datasets.

Three-dimensional point cloud data often contain spatial information from non-scanned objects, which may include unreasonable laser reflectance intensity (I-value) data. The boxplot method was applied to detect and filter out unreasonable, anomalous I-values in the laser reflectance intensity data for the three materials, ensuring a more accurate and reliable dataset for regression curve modeling [10]. These calculations were then represented graphically to show the data’s distribution and symmetry while also highlighting outliers. This visualization aided in the analysis and interpretation of the dataset, making the boxplot an effective data visualization method (Figure 2a) [11].

Boxplot analysis was applied to filter outliers in the classification data, which is critical for improving the accuracy of the classification model. The I-value range for each material was effectively defined by analyzing the I-values of wood, stone, and brick using boxplots, providing accurate data for constructing subsequent regression curves. Additionally, the visual nature of the boxplots enhanced the interpretability of classification benchmarks, aiding in identifying the distinctions between the materials.

This study utilized ground-based LiDAR to classify wood, stone, and brick materials commonly used in Minnan traditional architecture. The laser reflectance intensity (I-value) of the point cloud data for these three materials was represented via boxplots, allowing for visual analysis of the I-value characteristics and the relationships between the materials. Furthermore, the differences in the median values within the boxplots were the basis for the classification framework, ultimately achieving effective material classification.

### 2.4. Regression Analysis

Regression analysis is a statistical method used to demonstrate the relationship between two or more variables, typically presented in graphical form, and to derive a functional relationship between dependent and independent variables. Under ideal conditions, regression analysis can use one or more variables to predict another variable. However, in practical applications, regression analysis can only estimate the means or expected values of the variables [12,13].

The predictor variable *X* influences the tendency of the response variable *Y* to change statistically; the data points are distributed around the curve of the statistical relationship [14]. For different values of *X*, a probability distribution of the corresponding response variable *Y* can be determined. This relationship can be expressed concretely in the form of a function, referred to as the regression function of *Y* on *X*.

When this regression function is plotted, it forms a regression curve. Depending on the characteristics of the relationship, either a linear or a non-linear regression model can be chosen to construct the regression curve.

Due to the influence of multiple complex factors on the laser reflectance intensity (I-value), this study applied regression analysis theory to construct a regression model relating the I-value to the ground-based LiDAR scanning distance. A non-linear regression model was chosen for data analysis to capture the relationship between these two variables. However, increasing the degree of higher-order terms does not always improve the fit; excessive higher-order terms may distort the prediction results. The equation for the non-linear regression model used in this study is expressed as Equation (2).

(2) $$Y=\beta_{0}+\beta_{1}X+\beta_{2}X^{2}+\beta_{3}X^{3}+\ldots +\beta_{m}X^{m}+\varepsilon$$

Here,

*Y*: reaction variables;

$\beta_{0},\beta_{1},\beta_{2},\beta_{3}\ldots \beta_{n}$: coefficients of the regression model;

$\beta_{0}$: constant term;

$X,X^{2},X^{3}{\ldots X}^{n}$: different powers of the predictor variable;

ε: error term, representing the difference between the actual and predicted values of the model.

Equation (1) highlights three factors that influence the laser reflectance intensity (I-value): reflectivity (ρ), laser incidence angle (θ), and distance (R). However, Equation (2) simplifies the relationship by focusing on distance as the primary variable. Additional analyses can address this discrepancy by integrating the effects of the incidence angle (θ) and surface characteristics (e.g., roughness and reflectivity). These factors can be represented as auxiliary terms or parameters in a multivariable regression model. Future work should include experiments to quantify their specific contributions to the I-value and refine Equation (2) accordingly.

### 2.5. Experimental Design

The operation of ground-based LiDAR is highly susceptible to environmental factors, such as uneven lighting, temperature, and humidity. Additionally, the laser reflectance intensity (I-value) in point cloud data is inevitably influenced by physical phenomena, such as the scanning distance, incidence angle, scattering angle, and surface material of the scanned object. Thus, the I-value reflects the combined effects of these factors [15,16].

To address this, this study was structured into two phases:

1. Indoor experiment: this phase minimized environmental and physical influencing factors to analyze the characteristics of the I-value.
2. Field verification: the results from the indoor experiment were applied in outdoor scenarios to validate the classification method under real-world conditions.

The indoor experiment was conducted by establishing a controlled scanning environment with identical conditions. Data collection focused on a single material under the constraints of varying distances, uniform lighting, consistent incidence angles, and scattering angles. The findings from the indoor experiment were then used for verification in real-world cases to evaluate the effectiveness of the classification method.

Point cloud data collected from both the indoor experiment and field verification were subsequently processed using the boxplot method to filter out I-value outliers. The effective I-value ranges for the three materials were then compared. Regression curves incorporating the physical attributes of the three materials were constructed, and these regression models were ultimately used to achieve the effective classification of mixed materials.

**Figure 3:**
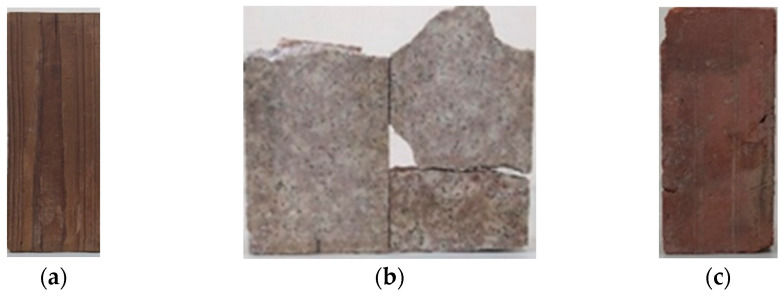
The materials used in the indoor experiment. (**a**) Wood; (**b**) stone; (**c**) brick.

This study focused on three materials commonly used in Minnan traditional architecture: wood, stone, and brick. All materials were sourced from actual Minnan traditional architecture to better simulate a realistic classification model. Relevant information about the materials used in the indoor experiment is shown in Figure 3.

The indoor environment minimized the influence of environmental and physical factors, enhancing the correlation between the laser reflectance intensity (I-value) and scanning distance for each material. Specifically, as the scanning distance increased, the I-value decreased; conversely, as the scanning distance decreased, the I-value increased. However, the surface characteristics of the materials still affected the I-value, resulting in a range of distributed values.

**Figure 4:**
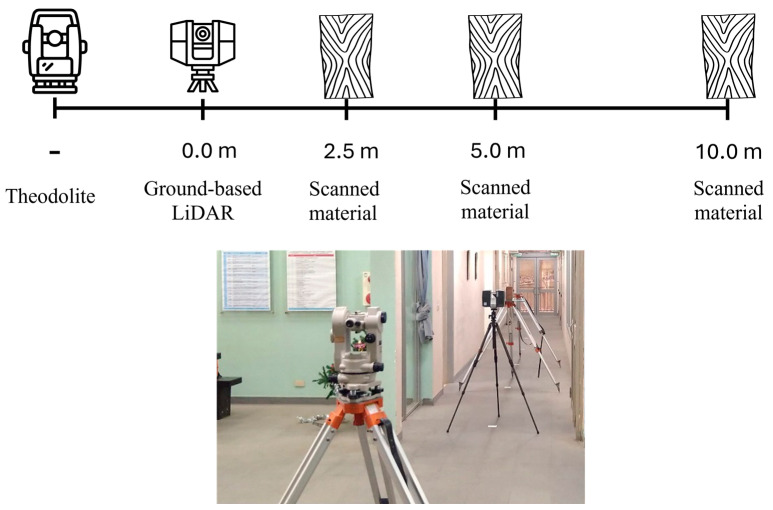
Schematic diagram of indoor experimental site layout and experiment overview.

During actual operations, point cloud data are collected from various scanning distances. To simulate this scenario, the experiment was designed to scan the same material at different distances to obtain data closely resembling real-world conditions. The layout of the indoor experimental site and a summary of the experimental setup are shown in Figure 4.

For each material, scanning was conducted at distances of 2.5 m, 5 m, and 10 m from the ground-based LiDAR. A theodolite was used to ensure consistency in the laser incidence angle.

## 3. Results

### 3.1. Indoor Experiment Results

**Figure 5:**
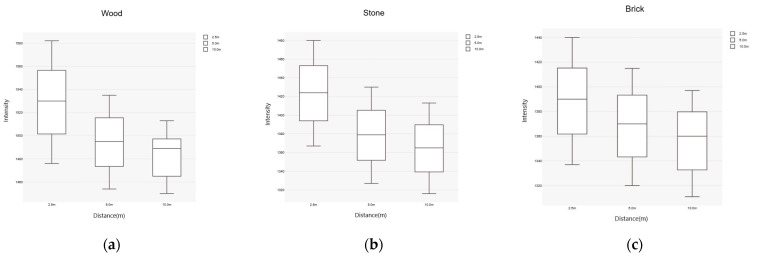
Boxplots of the three materials. (**a**) Wood; (**b**) stone; (**c**) brick.

The laser reflectance intensity (I-value) of the three materials obtained at different scanning distances was used to create boxplots illustrating the relationship between scanning distance and I-value, as shown in Figure 5. A comprehensive review and analysis of the boxplots for the three materials revealed the following characteristics:

- The I-values for wood, stone, and brick exhibited continuity across different scanning distances.
- The box lengths of the boxplots for the same material remained similar across different distances.
- The I-values were concentrated within the 50% range between quartiles 2 and 3 of the boxplots, with relatively consistent lengths, indicating that the collected data were less influenced by environmental and physical factors.

The characteristics of the point cloud data after scanning are shown in Table 1, Table 2 and Table 3. As the scanning distance increased, the total number of point cloud coordinates decreased. After removing outliers using the boxplot method, the I-values exhibited a reasonable distribution from large to small as the scanning distance increased. The $\bar{I}$-value represents the average of the I-values.

**Figure 6:**
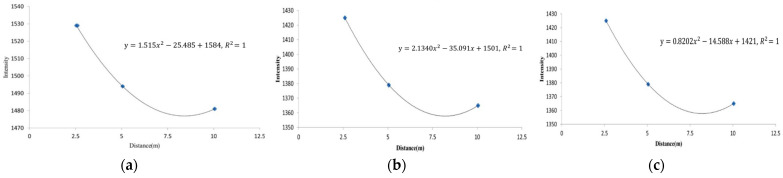
Polynomial regression curves for the three materials. (**a**) Wood; (**b**) stone; (**c**) brick.

Based on Table 1, a quadratic polynomial regression model was used to fit the relationship between the average laser reflectance intensity (I-value) and different scanning distances. The resulting regression curve is shown in Figure 6, where the coefficient of determination R^2^ indicates a high degree of curve fitting accuracy.

After constructing the regression curves for the three materials, these curves inherently included the attributes influenced by environmental and physical factors, making them material-specific equations. These regression curves were the basis for classifying the three materials.

A unique identification code was added to each point cloud coordinate of the three materials to evaluate the classification effectiveness. The format of the annotated data was (A0001, X, Y, Z, I), where “A0001” represents the first coordinate in the wood point cloud dataset, followed sequentially by A0002, A0003, …. Similarly, “B0001” represents the first coordinate in the stone dataset, followed by B0002, B0003, …, and “C0001” represents the first coordinate in the brick dataset, followed by C0002, C0003, …. The combined point cloud data with annotated identification codes for the three materials became the test file for the classification experiments. Subsequently, the distance variable from the point cloud coordinates to the LiDAR instrument center was inputted into the regression equations of the three materials. This yielded the calculated laser reflectance intensity $\hat{I}$, which was expected to closely match the actual I-value, such that $∆I=I-\hat{I}$, where ∆I approaches zero. For classification, the absolute value of ∆I, denoted by |∆I|, was calculated. If |∆I| for a specific coordinate approached its minimum when tested against the regression equation of a material, that coordinate was classified as belonging to the point cloud group of that material. For example, the coordinates with the smallest |∆I| values derived from the wood regression equation were classified as wood using the regression equation for wood to test the mixed point cloud dataset.

**Figure 7:**
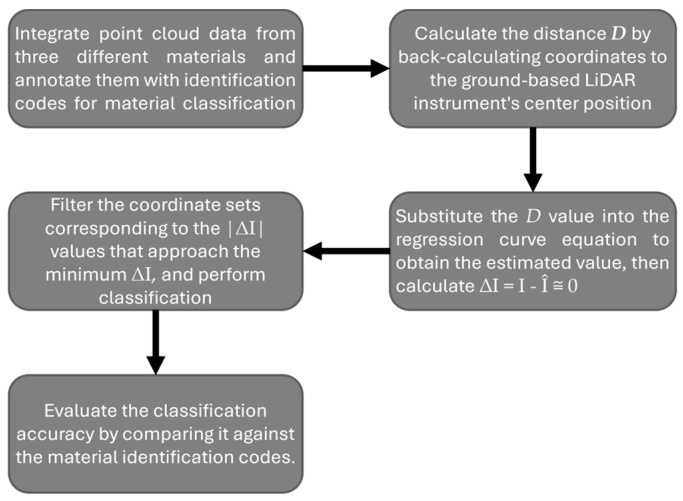
Proposed classification process diagram.

The classification accuracy was verified by comparing the material identification codes added to each coordinate, which served as indicators for the classification consistency. The developed classification process is illustrated in Figure 7.

Based on the previous content and the classification process diagram in Figure 7, classification tests were conducted for the three materials, yielding the results shown in Table 4. The classification accuracy was 95% for wood and stone, and 99% for brick. These results indicate that the classification process and methods developed from the indoor experiment can be effectively applied to real-world scenarios.

The analysis revealed that the classification accuracy of wood (74%) was lower than that of the other materials (stone: 98%; brick: 93%). This discrepancy may have stemmed from the lower surface roughness of wood, resulting in less-stable variations in the laser reflectance intensity (I-value), especially in long-range scans. Additionally, the lower accuracy might relate to the model’s performance in handling minor differences in I-values. The regression model should be optimized further to enhance the classification effectiveness for wood.

### 3.2. Field Verification

Reference point clouds were carefully selected based on specific criteria to validate the classification results, particularly for outdoor scenes. Outdoor data were collected under controlled weather conditions (clear and sunny days) to minimize environmental variability. Sampled reference points were chosen from real-world structures representative of traditional Minnan architecture, specifically stone, wood, and brick materials. These materials were scanned at multiple distances (e.g., 3 m, 6 m, and 9 m) to capture variations caused by scanning angles and distances. The data collected were then cross verified using manual annotation to ensure accuracy and reliability. This process ensured that the reference datasets provided a robust basis for assessing the classification accuracy of the proposed method.

**Figure 8:**
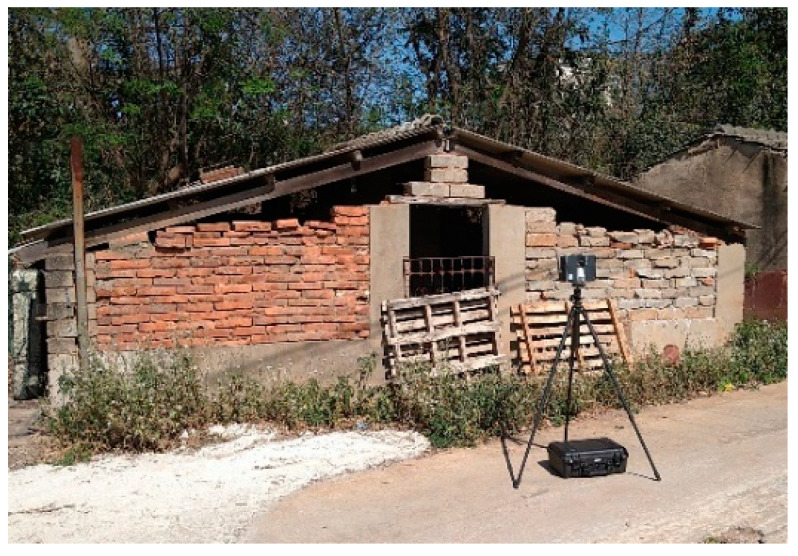
Wall surfaces with three types of traditional Minnan architectural materials.

A wall constructed with wood, stone, and brick—three materials commonly used in Minnan traditional architecture—on Kinmen Island was selected as the verification case (Figure 8). The FARO Focus 3D ground-based LiDAR was positioned at three distances from the wall: approximately 3 m (close range), 6 m (medium range), and 9 m (long range). Scanning operations were conducted under clear and sunny weather conditions with adequate lighting for point cloud data collection.

The indoor experiments achieved a classification accuracy of 95% to 99% under the premise of controlled environmental and physical factors. However, in practical applications, ground-based LiDAR operations cannot replicate the controlled environmental and physical conditions of laboratory settings. Therefore, the acquisition of sample data for regression curve modeling must be carefully planned in advance.

Based on the experience of indoor experiments, point cloud data should be obtained for the same material at different scanning distances to match the spatial relationship between the scanning station and the scanned object during actual ground-based LiDAR operations.

**Figure 9:**
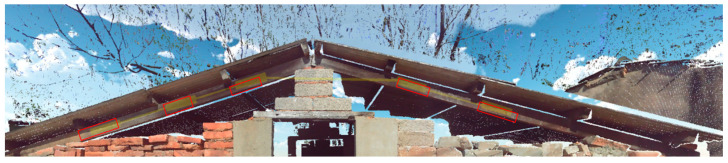
Regression data sampling diagram (wood).

**Figure 10:**
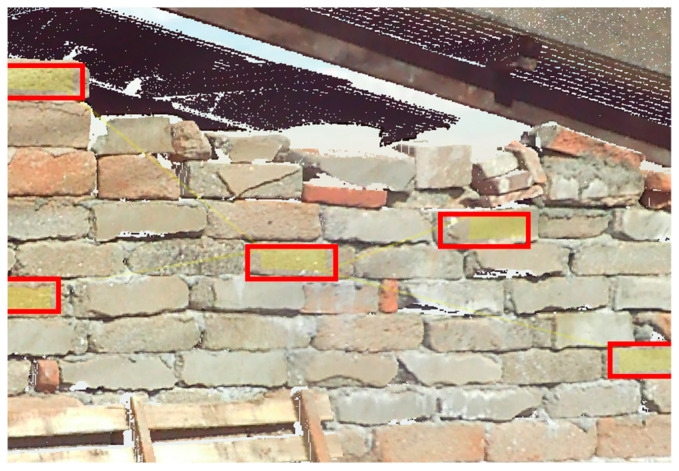
Regression data sampling diagram (stone).

**Figure 11:**
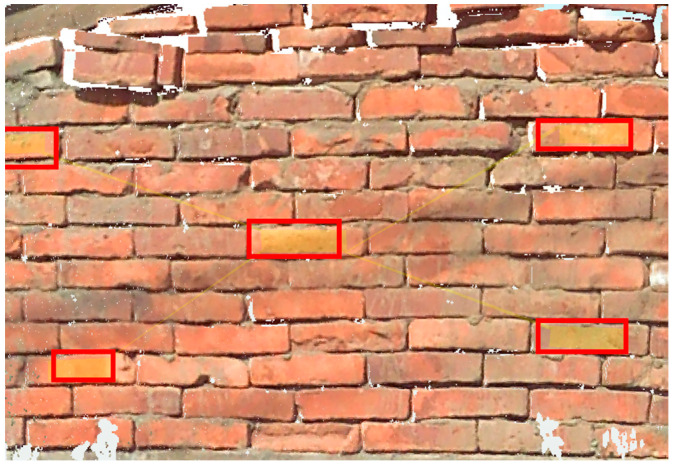
Regression data sampling diagram (brick).

The sampling method involved uniformly acquiring sample data for the three types of materials. Point cloud data from three scanning positions were used for sampling. Figure 9, Figure 10 and Figure 11 demonstrate the sampling case using a scanning distance of 3–4 m, with the red box indicating the sampling area.

The sampled point cloud data for the three materials were each calibrated using boxplots to detect outliers in the laser reflection intensity I-values for each material. As shown in Table 5, all three materials followed the general rule that the further the scanning distance, the fewer the number of acquired point clouds. However, the I-values for the three materials did not adhere to the indoor experimental rule that I-values decrease as the scanning distance increases. This discrepancy was attributed to the effects of uneven outdoor lighting, inconsistent incidence angles, and scattering angles.

This observation indicates that environmental and physical factors directly influence the laser reflection intensity I-values. By employing polynomial regression curve modeling, these influencing factors can be incorporated into the simulation equation. Since the sampled point cloud data reflect variations due to different scanning distances and positions (incidence and scattering angles), the I-values inherently capture these influencing factors.

Therefore, the regression curve constructed from the sampled data in this study can be regarded as a regression equation that encapsulates the degree of influence caused by the environmental and physical factors present during scanning operations.

**Figure 12:**
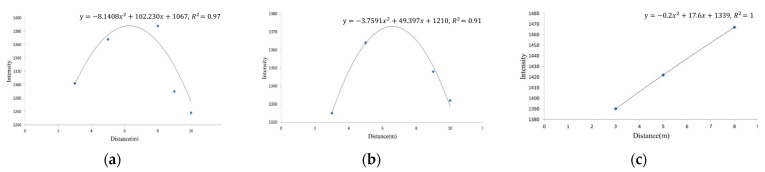
Polynomial regression curve chart for the three sampled materials. (**a**) Wood; (**b**) stone; (**c**) brick.

The sampled data were used to construct a regression curve based on the classification procedure developed in Figure 7 and the indoor experiment content. The resulting regression curve and its equation, as shown in Figure 12, clearly differ in trend and linearity from those obtained in indoor experiments. This indicates that the regression results inherently include the effects of environmental and physical factors.

Three wall point cloud datasets obtained from three different scanning distances were manually extracted to identify the three materials—wood, stone, and brick—as the baseline for assessing and verifying the classification accuracy. Figure 11 provides an example using point cloud data from the 3 m scanning position.

Classification tests were conducted for the three-point cloud datasets from the three positions following the classification procedure developed in Figure 7 and the indoor experimental approach. The classification results are shown in Table 6. According to Table 6, the classification accuracy for wood at the 9 m scanning distance was 54%, with an overall average accuracy of 74%, which was lower than the values of 98% for stone and 93% for brick. This discrepancy was attributed to the lower surface roughness of wood and the larger scanning tilt angle. However, the classification accuracy for all three materials reached over 98% at a scanning distance of 6 m.

**Figure 13:**
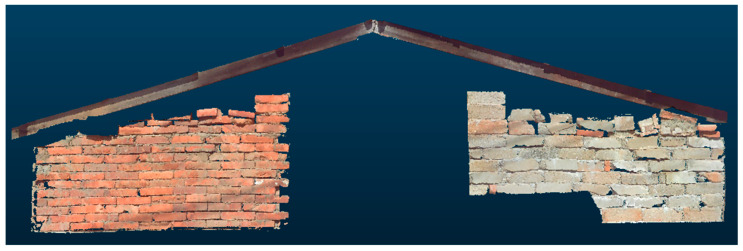
Regression data sampling diagram (brick).

**Figure 14:**
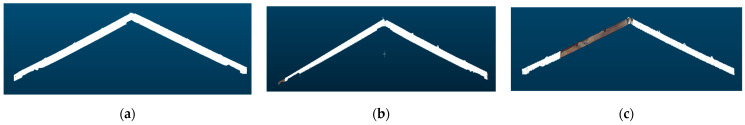
Overlay differences before and after point cloud classification (wood): (**a**) 3 m; (**b**) 6 m; (**c**) 9 m.

**Figure 15:**
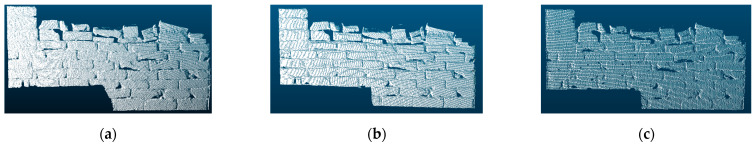
Overlay differences before and after point cloud classification (stone): (**a**) 3 m; (**b**) 6 m; (**c**) 9 m.

**Figure 16:**
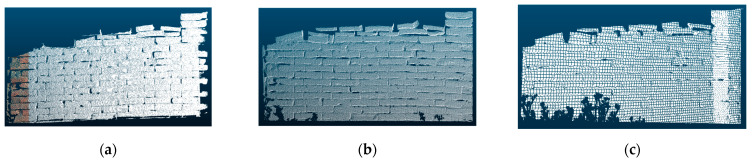
Overlay differences before and after point cloud classification (brick): (**a**) 3 m; (**b**) 6 m; (**c**) 9 m.

As presented in Table 6, the classification accuracy at varying distances demonstrates significant differences, particularly at 3 m and 9 m, compared with the accuracy at 6 m. This discrepancy can be attributed to environmental and physical factors influencing the laser reflectance intensity values. Specifically, at 3 m, the classification accuracy was likely impacted by a high scanning tilt angle, which increased reflection inconsistencies for materials such as wood. At 9 m, the longer scanning distance introduced more scattering effects and reduced signal strength, leading to lower accuracy. In contrast, the accuracy at 6 m was significantly higher, achieving over 98% for all materials. This result aligns with the optimal conditions identified during indoor experiments, where medium distances minimized environmental interferences and discrepancies caused by physical angles. See Figure 13, Figure 14, Figure 15 and Figure 16.

Furthermore, as summarized in Table 6 for stone and brick materials at various distances, a comparison reveals that wood is more susceptible to environmental variations due to its surface roughness and reflective properties. These variations highlight the necessity of refined calibration models to improve classification consistency across different distances.

## 4. Discussion

The experimental results demonstrate that the classification accuracies for wood, stone, and brick were 74%, 98%, and 93%, respectively. While these results are promising, they highlight areas where the proposed method could be refined to enhance its accuracy and applicability. Existing point cloud classification methods, which often rely on manual visual selection or geometric features, face limitations in handling complex datasets, especially under varying environmental conditions. The integration of advanced machine learning techniques, such as neural networks or support vector machines, could complement the current framework by enabling more effective feature extraction and reducing the impact of environmental noise.

A detailed analysis of the experimental results revealed several key insights. The comparatively lower classification accuracy for wood (74%) was likely due to the smoother surface texture of wood, which affected the consistency of the laser reflectance intensity (I-value) across varying scanning distances. Additionally, the larger scanning tilt angles for wood resulted in higher variability in the I-values, which may have reduced the effectiveness of the regression model. Addressing these challenges might involve optimizing the scanning setup, such as minimizing tilt angles or employing preprocessing techniques, such as I-value normalization, to improve consistency.

The classification accuracy of stone reached the highest level (98%), possibly due to its uniform surface characteristics and higher laser reflectivity. The I-value range of stone demonstrated stability across varying scanning distances, allowing the model to distinguish stone more accurately from other materials. Furthermore, this suggests that the physical properties of stone are less sensitive to environmental and geometric conditions, contributing to the robustness of the classification model for stone.

Another significant factor was the influence of environmental conditions during outdoor scanning, which may have introduced additional inconsistencies compared with the controlled indoor conditions. Factors such as uneven lighting, variable humidity, and inconsistent incidence angles contributed to fluctuations in the I-values, impacting the reliability of the classification. Future research could explore adaptive correction algorithms to account for environmental variables, further improving the robustness and accuracy of the proposed classification framework.

The proposed method also addressed the issue of significant overlap in I-value ranges between different materials by leveraging polynomial regression curve equations. These equations effectively model the relationship between I-values and scanning distances, providing a robust basis for classifying materials with similar properties. Despite these innovations, integrating complementary approaches, such as combining geometric and reflectance data or employing clustering algorithms, could further enhance the classification accuracy and expand the framework’s applicability.

## 5. Conclusions

The experimental results demonstrate that the classification accuracies for wood, stone, and brick were 74%, 98%, and 93%, respectively. While these results are promising, the accuracy and applicability of the proposed method can be enhanced. Existing point cloud classification methods, which often rely on manual visual selection or geometric features, face limitations in handling complex datasets. Incorporating advanced machine learning techniques, such as neural networks or support vector machines, could complement the current approach by enabling better feature extraction and reducing the influence of environmental noise.

Furthermore, a more thorough analysis of the experimental results revealed several key insights. First, the lower classification accuracy for wood (74%) compared with stone (98%) and brick (93%) can be attributed to the smoother surface of wood, which reduced the reflectance intensity (I-value) consistency across the varying scanning distances. Additionally, the larger scanning tilt angles for wood contributed to the higher variability in the I-values. Addressing these challenges might involve optimizing the scanning setup, such as by reducing the tilt angle or employing preprocessing techniques to normalize the I-values.

Another significant factor was the influence of environmental conditions during outdoor scanning, which may have introduced inconsistencies into the results compared with the controlled indoor experiments. Future studies could focus on improving robustness by incorporating adaptive correction algorithms that account for environmental variables such as lighting and humidity.

In summary, while the proposed method offers a robust framework for automated material classification, integrating complementary approaches and refining experimental setups could further enhance its accuracy and adaptability for real-world applications. This would also bridge the gap between laboratory experiments and practical implementations, expanding the method’s usability in diverse scenarios, such as heritage preservation, urban planning, and environmental monitoring.

## Figures and Tables

**Table 1:** Characteristics of point cloud data after scanning (wood).

Wood	Point Cloud	I-Value	$\bar{I}$-Value
2.5 m	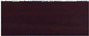	1476–1582	1529
5.0 m	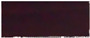	1454–1535	1494
10.0 m	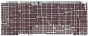	1450–1513	1481

**Table 2:** Characteristics of point cloud data after scanning (stone).

Stone	Point Cloud	I-Value	$\bar{I}$-Value
2.5 m	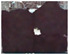	1367–1480	1425
5.0 m	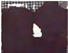	1327–1430	1379
10.0 m	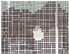	1316–1413	1368

**Table 3:** Characteristics of point cloud data after scanning (brick).

Brick	Point Cloud	I-Value	$\bar{I}$-Value
2.5 m	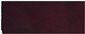	1337–1440	1390
5.0 m	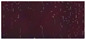	1320–1415	1369
10.0 m	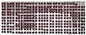	1311–1397	1358

**Table 4:** Indoor experiment classification results.

Classification Objectives	Distribution Range of $\hat{I}$ from *D* in Regression Equation	Distribution Range of Point Cloud I-Value	Average Laser Reflection Intensity $\bar{I}$-Value of I	Classification Indicator $∆I\;=\;I-\hat{I}$	Number of Point Clouds After Classification	Number of Correctly Classified Point Clouds Verified	Classification Accuracy Percentage
Wood	1481–1529	1450–1582	1522	≅0	21,742	20,744	95%
Stone	135–1425	1316–1480	1413	≅0	14,817	13,995	95%
Brick	1358–1390	1311–1440	1385	≅0	8614	8714	99%

**Table 5:** Characteristics of point cloud data after scanning.

Material	Distance	3.0	5.0	8.0	9.0	10.0
Wood	I-value	1235~1376	1285~1452	1179~1530	1250~1332	1228~1288
	$\bar{I}$-value	1302	1368	1388	1290	1258
Stone	I-value	1270~1379	1299~1431	-	1303~1394	1297~1366
	$\bar{I}$-value	1325	1364	-	1348	1332
Brick	I-value	1287~1490	1296~1503	1333~1558	-	-
	$\bar{I}$-value	1390	1422	1467	-	-

**Table 6:** Practical validation of classification accuracy for three materials.

Material	Wood			Stone			Brick		
Distance	3.0	6.0	9.0	3.0	6.0	9.0	3.0	6.0	9.0
Original point cloud quantity	164,597	57,373	38,661	314,522	158,167	41,620	434,007	196,198	84,691
Point cloud quantity after classification	232,555	56,489	20,944	330,185	156,111	41,320	354,149	193,255	83,590
Classification accuracy	71%	98%	54%	95%	99%	99%	82%	99%	99%
Average classification accuracy	74%			98%			93%		

## Data Availability

Data are contained within the article.

## References

[1] Akca D. (2007). Matching of 3D surfaces and their intensities. J. Photogramm. Remote Sens..

[2] Song J.-H., Han S.-H., Yu K., Kim Y.-I. (2002). Assessing the Possibility of Land-Cover Classification Using Lidar Intensity Data. Int. Arch. Photogramm. Remote Sens. Spat. Inf. Sci..

[3] Vain A., Kaasalainen S. (2010). Correcting Airborne Laser Scanning Intensity Data for Automatic Gain Control Effect. IEEE Geosci. Remote Sens. Lett..

[4] Chow J.C. (2014). Multi-Sensor Integration for Indoor 3D Reconstruction. Ph.D. Dissertation.

[5] FARO (2013). FARO Focus 3D, Features, Benefits & Technical Specifications.

[6] FARO (2013). FARO Focus 3D, FARO Laser Scanner 3D User Manual.

[7] Höfle B., Pfeifer N. (2007). Correction of laser scanning intensity data: Data and model-driven approaches. ISPRS J. Photogramm. Remote Sens..

[8] Tan K., Cheng X. (2016). Correction of incidence angle and distance effects on TLS intensity data based on reference targets. Remote Sens..

[9] Kashani A.G., Olsen M.J., Parrish C.E., Wilson N. (2015). A review of LiDAR radiometric processing: From Ad Hoc intensity correction to rigorous radiometric calibration. Sensors.

[10] Pulikkaseril C., Ross D., Tofini A., Lize Y.K., Collarte F. (2024). Material Classification Using a Polarization-Diverse RMCW LIDAR. Sensors.

[11] Williamson D.F., Parker R.A., Kendrick J.S. (1989). The box plot: A simple visual method to interpret data. Ann. Intern. Med..

[12] Adams N.M., Blunt G., Hand D.J., Kelly M.G. (2000). Data Mining for Fun and Profit. Stat. Sci..

[13] Chai T., Draxler R.R. (2014). Root mean square error (RMSE) or mean absolute error (MAE). Geosci. Model Dev. Discuss..

[14] Newcastle University Online Resources. https://www.ncl.ac.uk/webtemplate/ask-assets/external/maths-resources/.

[15] Guidi G., Malik U.S., Manes A., Fossati S.C.M., Lazzari C., Volpato C., Giglio M. (2020). Laser Scanner-Based 3D Digitization for the Reflective Shape Measurement of a Parabolic Trough Collector. Energies.

[16] Tan K., Cheng X. (2017). Specular Reflection Effects Elimination in Terrestrial Laser Scanning Intensity Data Using Phong Model. Remote Sens..

